# CEMP-1 Levels in Periodontal Wound Fluid during the Early Phase of Healing: Prospective Clinical Trial

**DOI:** 10.1155/2019/1737306

**Published:** 2019-02-24

**Authors:** Claudia Dellavia, Elena Canciani, Giulio Rasperini, Giorgio Pagni, Matteo Malvezzi, Gaia Pellegrini

**Affiliations:** ^1^Department of Biomedical, Surgical and Dental Sciences, Università degli Studi di Milano, Via Mangiagalli 31, 20133 Milan, Italy; ^2^Foundation IRCCS Ca' Granda Ospedale Maggiore Policlinico, Via della Commenda 10, 20122 Milan, Italy; ^3^Department of Clinical Sciences and Community Health, Università degli Studi di Milano, Via Vanzetti 5, 20133 Milan, Italy

## Abstract

**Objectives:**

Cementogenesis seems to be significantly compromised during tissue inflammation. In dental practice, surgical procedures are performed with the aim to regenerate periodontium including cementum. However, inflammation that occurs during the initial healing phases after surgery may impair regeneration of this tissues. The aim of the present study was to assess if surgical procedures designed to regenerate periodontium might affect levels of cementum protein-1 (CEMP-1) in periodontal wound fluid during early phase of healing.

**Materials and Methods:**

In 36 patients, 18 intrabony periodontal defects were treated with regenerative therapy (REG group) and 18 suprabony periodontal defects were treated with open flap debridement (OFD group). In the experimental sites, gingival crevicular fluid was collected immediately before surgery, and periodontal wound fluid was collected 4, 7, 14, and 21 days after surgery. CEMP-1 levels were detected by indirect enzyme-linked immunosorbent assay technique.

**Results:**

At the analysis, it resulted that there was a significant average difference in CEMP-1 values between the REG and OFD groups at baseline (*p* = 0.041), the CEMP-1-modeled average in the OFD group was lower by 0.45 ng/ml. There was a significant trend in CEMP-1 over time, and this trend was different among the 2 groups: the REG group showed a statistically significant rising CEMP-1 trend (0.18 ng/ml a week *p* = 0.012), while the OFD had a trend that was significantly lower (-0.22 ng/ml a week compared to the REG group trend *p* = 0.023), the OFD group lost on average 0.05 ng/ml a week. In REG sites, GCF protein levels resulted also related to clinical parameters.

**Conclusions:**

During the initial inflammatory phase of periodontal healing, CEMP-1 levels decrease regardless of the surgical protocol applied. The surgical procedures used to regenerate periodontal tissue are able to reverse this trend and to induce significant increase of CEMP-1 in periodontal wound fluid after the first week postop.

## 1. Introduction

Gingival crevicular fluid (GCF) is a physiological serum transudate that flows through the junctional epithelium to gingival sulcus and that can be collected at the gingival margin or within the gingival crevice. This fluid is called periodontal wound fluid (PWF) when it is derived from postsurgical healing sites. After periodontal surgical trauma, cell-signaling protein molecules (e.g., growth factors, chemokines, or cytokines) and products of cellular activity (enzymes and adhesion molecules) are released in the wound-healing area. Levels of cytokines, chemokines, and angiogenic biomarkers within the gingival crevicular fluid and in periodontal wound fluid have been studied in clinical trials to assess the ongoing angiogenesis, connective tissue, and bone formation activities during wound-healing phases (inflammation, granulation tissue formation, and tissue neoformation/remodeling) [1, 2]. Morelli et al. [3] evaluated changes of angiogenic markers in wound fluid after placement of a soft tissue autograft or of a living cellular construct for treatment of mucogingival defects. The authors observed that these procedures heal in two different ways and that levels of the analyzed biomarkers also resulted different. Eren et al. [4] characterized the wound-healing activity and inflammation of localized gingival recession defects treated with coronally advanced flap plus platelet-rich fibrin compared with coronally advanced flap plus connective tissue graft, and they observed that platelet-rich fibrin may promote early wound healing by elevating the levels of tissue inhibitor of matrix metalloproteinases-1 and suppressing the levels of proinflammatory and remodeling molecules (matrix metalloproteinase-8 and IL-1b) in gingival crevicular fluid at 10 days after surgery. Pellegrini et al. [5] assessed the levels of proteins related to epithelium, connective tissue, and bone healing as possible biological indicators of clinical outcome at 6 months after surgery. Comprehension of physiology of cementum and related molecules is important for the development of potential new therapies in periodontal regeneration. However, clinical studies evaluating specific markers for cementum activity are lacking. Cementum protein-1 (CEMP-1) is a tissue-specific protein for cementum. This protein is only expressed by cementoblasts, their progenitors, and by periodontal ligament-derived cells [6, 7]. CEMP-1 has been detected lining the cementum surface, in the perivascular area and within the periodontal ligament throughout the root surface [6]. One in vitro study observed that CEMP-1 stimulates migration and proliferation of periodontal ligament cells and promotes cell differentiation, maturation, and deposition of mineralized extracellular matrix resembling cementum [7]. It also reduces the level of osteoblastic markers and increases the amount of cementoblastic markers. Furthermore, overexpression of CEMP-1 was found to slightly increase cementogenesis and differentiation of cementoblasts [7]. The levels of CEMP-1 reduce significantly after stimulation of cementoblasts with IL-1*β*, and cementogenesis may be significantly compromised during tissue inflammation [8]. In dental practice, surgical procedures are performed with the aim to regenerate periodontium including cementum, ligament, and alveolar bone. However, inflammation that occurs during the initial healing phases after surgery may impair this tissue regenerative activity.

The aim of the present study was to assess if surgical procedures designed to regenerate periodontium might affect levels of CEMP-1 in periodontal wound fluid during initial healing.

## 2. Materials and Methods

This is a prospective clinical trial. The study has been approved by the ethical committee of Università degli Studi di Milano (Italy) (18.10.11, number 30/11). All procedures performed in the studies involving human participants were in accordance with the ethical standards of the institutional and/or national research committee and with the 1964 Helsinki Declaration and its later amendments or comparable ethical standards.

### 2.1. Study Population

A total of 36 volunteers were enrolled: 18 patients that had an intrabony periodontal defect requiring regenerative therapy (REG group) and 18 patients that had a horizontal periodontal defect (without intrabony components) requiring open flap debridement (OFD group). Enrolled patients presented the following inclusion criteria:

- age range: 25-80 years old
- nonsmoking (former smokers were included if they had not smoked within 6 months of the study initiation)
- suffering from periodontitis at stage 3 grade B [9] and completed the initial periodontal therapy from at least 3 months
- defect anatomy OFD group: presence of at least one tooth with probing pocket depth (>5 mm) and clinical attachment level with an intrabony defect ≤ 3 mm
- defect anatomy REG group: presence of at least one tooth with probing pocket depth (>5 mm) and clinical attachment level of ≥5 mm associated with an intrabony defect of >3 mm
- good oral hygiene: full mouth plaque and bleeding scores ≤ 20% at the beginning of the study (baseline)
- experimental teeth had to be vital or properly treated with root canal therapy
- subgingival margins, open margins, overhanging margins, and inadequate restorations had to be absent in the experimental site

The following exclusion criteria were applied:

- patients with clinically significant or unstable organic diseases, immune-compromised, taking steroid medications, chronically treated (i.e., two weeks or more) with any medication known to affect periodontal status (i.e., antibiotics or nonsteroidal anti-inflammatory drugs), and taking antibiotics within 90 days of baseline
- patients displaying compromised healing potential such as those affected by connective tissue disorders or bone metabolic diseases; patients with conditions requiring antibiotic prophylaxis
- pregnant or lactating women, or women who were of childbearing potential and not using birth control or abstinence
- current smokers or former smokers who had smoked in the previous 6 months
- patients affected by active infectious diseases

### 2.2. Study Procedures

**Figure 1:**
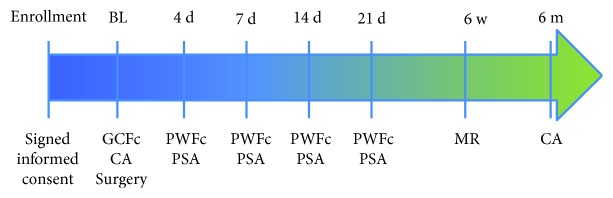
Timeline of the study. GCFc: gingival crevicular fluid collection; CA: clinical assessments (probing pocket depth, clinical attachment level, full mouth plaque and bleeding, photographs, X-ray); PWFc: periodontal wound fluid collection; PSA: postsurgical clinical assessments of healing; BL: baseline; d: days; MR: membrane removal.

The study timeline is reported in Figure 1. After verification of the inclusion/exclusion criteria and sign of the informed consent, each patient was enrolled into the study. Gingival crevicular fluid was harvested and clinical measurements and radiographs were taken (baseline). Before the surgical appointment, all patients underwent professional oral hygiene procedure and instructions were given to eliminate any infective complications. Each patient's compliance to the experimental protocol was also confirmed.

In both groups (open flap debridement and regenerative therapy), local anesthesia with mepivacaine 2% 1 : 100.000 epinephrine was administered. In all the sites (OFD and REG), full-thickness flap was incised and elevated.

**Figure 2:**
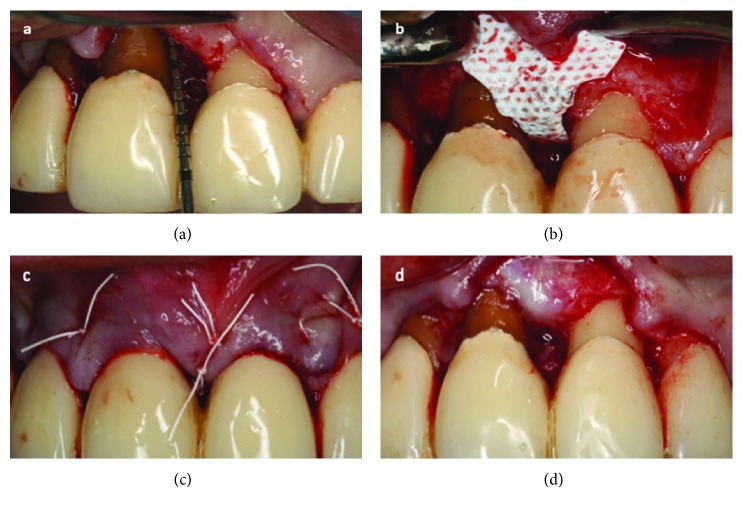
In the sites with intrabony periodontal component, after flap elevation, the granulation tissue was removed and roots were planned (a). The defect was covered with nonresorbable titanium-reinforced completely inert membrane (dense polytetrafluoroethylene) alone with no bone substitutes (b) and closed with a single-modified internal mattress suture (c). The membrane was removed 6 weeks after surgery (d).

In the 18 REG sites, the simplified papilla preservation technique was adopted whenever the width of the interdental space was 2 mm or narrower, while the modified papilla preservation technique was applied when the interdental sites were wider than 2 mm [10, 11]. The intrasulcular interdental incision (SPPF or MPPT) was extended to the buccal and lingual aspects of the mesial and distal teeth adjacent to the defect. After flap elevation, the granulation tissue was removed, and the intrabony defects were cleaned by hand instrumentation, ultrasonic scalers, and the root planning was done. Vertical releasing incisions were performed when flap reflection caused tension at the extremities of the flap(s). REG defects were covered with a nonresorbable titanium-reinforced completely inert membrane (dense polytetrafluoroethylene) (Cytoplast®, Osteogenics Biomedical, Lubbock, Texas, USA) alone with no bone substitutes and closed with a single modified internal mattress suture (polytetrafluoroethylene 6/0); thus, a tension-free primary closure of the papilla was obtained (Figure 2).

**Figure 3:**
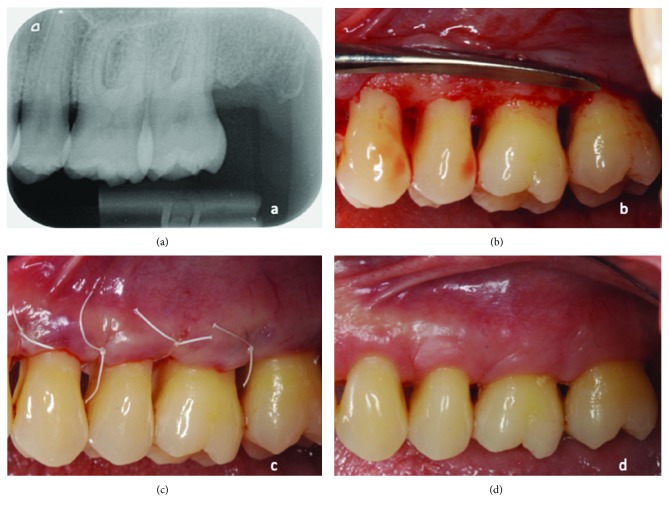
Modified Widman Flap was performed in sites with suprabony periodontal defects (a). After flap elevation, the granulation tissue was removed and roots were planned (b). Sites were closed with single external mattress suture (c). Sutures were removed 1 week after surgery (d).

In the OFD sites, modified Widman Flap was performed [12]. After flap elevation, the granulation tissue was removed, and the horizontal defects were cleaned by hand instrumentation, ultrasonic scalers, and the roots were planned. The OFD sites were closed with a single external mattress suture (polytetrafluoroethylene 5/0) (Figure 3).

Postoperative pain and edema were controlled with ibuprofen. Patients received 600 mg at the beginning of the surgical procedure and were instructed to take another tablet 6 hours later. Subsequent doses were taken only if necessary to control pain. Patients with ulcers, gastritis, and other contraindications to nonsteroidal anti-inflammatory drugs received 500 mg acetaminophen. All patients were instructed to intermittently apply an ice bag on the operated area (20 minutes per hour for 24 hours). All patients were instructed to discontinue tooth brushing and avoid trauma at the surgical site for a period of time ranging between 3 and 4 weeks. A 60-second rinse with 0.12% chlorhexidine digluconate was prescribed 3 times/day for the first 3 to 4 weeks.

At 4, 7, 14, and 21 days after surgery, periodontal wound fluid was collected by a blind operator (EC). Clinical measurements were taken 6 months after surgery.

### 2.3. Gingival Crevicular Fluid or Periodontal Wound Fluid Harvesting and Analysis

In all the patients, gingival crevicular fluid or periodontal wound fluid was collected from the study site at baseline, and 4, 7, 14, and 21 days after surgery (Figure 1) as previously reported [13]. Prior to crevicular fluid collection, supragingival plaque biofilm in the area around each sample was removed and the site was air-dried. Two methylcellulose paper strips (Periopaper®, ProFlow Inc., Amityville, NY) were gently inserted in the gingival sulcus or periodontal pocket for 1-2 mm. The fluid sample was then collected for 30 seconds, and the strips were placed into the Eppendorf tubes. The samples of gingival crevicular fluid and periodontal wound fluid were subsequently kept on dry ice and stored at -20°C until analysis.

Prior to biomarker analysis, gingival crevicular fluid and periodontal wound fluid were thawed at room temperature, and proteins were eluted with 100 *μ*L of 1× phosphate buffered saline solution and Protease Inhibitor PMSF (Thermo Fisher Scientific, Italy). To retrieve the crevicular fluid sample, the paper strips were centrifuged at 15 000×g for 5 min for 4 times, and the total volume of 100 *μ*L was obtained. The indirect enzyme-linked immunosorbent assay technique was used to detect and quantify levels of CEMP-1 protein (ng/ml) in the crevicular/wound fluid using the MBS702364 kit (MyBioSource) according to manufacturer's instruction. The indirect enzyme-linked immunosorbent assay test was performed measuring the absorbance at 450 nm by means of a spectrophotometer plate reader (Wallac Victor II plate reader).

### 2.4. Clinical and Radiographical Analysis

The following clinical measurements were taken at baseline and 6 months after surgery:

- full mouth plaque and bleeding score at the four sites for all teeth
- probing pocket depth, recession, and clinical attachment level (calculated as the sum of probing pocket depth and recession) were assessed at six sites of each tooth treated

Reduction of probing pocket depth and gain of clinical attachment level were calculated, respectively, as the difference between probing pocket depth or clinical attachment level at baseline and probing pocket depth or clinical attachment level at 6 months.

All measurements were taken with a UNC periodontal probe (Hu-Friedy Manufacturing Company Inc., Chicago, IL, USA).

Intraoral radiographs of the defect were taken using Rinn's attachment and a long cone parallel technique at baseline and 6 months after periodontal surgery.

Intraoral photographs of the experimental sites were taken before surgery, after defect debridement, after flap closure, at weeks 1, 2, and 3, and at 6 months.

### 2.5. Statistical Analysis

Each patient represented a statistical unit, and only one defect was treated for each volunteer. In both groups, mean and standard deviation were calculated for probing pocket depth (mm) and clinical attachment level (mm) at baseline and at 6 months postop and for levels of CEMP-1 (ng/ml) at baseline and 4, 7, 14, and 21 days after surgery.

Preliminary analysis was carried out at baseline measuring the difference in mean CEMP-1 (ng/ml) levels between the REG and OFD groups with a *t*-test. As a first evaluation, *t*-tests of average differences between CEMP-1 at baseline and treatment day 21st were carried out between the groups (i.e., REG and OFD); furthermore, *t*-tests for paired data were applied to both the REG and OFD groups between CEMP-1 levels at baseline and day 21st. These evaluations were only carried out between measurements at baseline and at day 21st to avoid pseudoreplication and multiple testing issues.

To properly characterize the data, a linear mixed model was used, in order to account for repeated measures on patients over time [14]. The study treatment group (REG vs OFD) and time (observation week) and their interaction to capture nonparallel growth trends were included as fixed effects; patients were considered as random effects in particular with respect to time (days). Finally, since the same patients were measured over time, a first order autocorrelation structure was used. The nlme library from the R-project statistical suit was used to fit the linear mixed model. A level of significance of 5% (*p* < 0.05) was considered.

## 3. Results

A total of 36 nonsmoker patients, 18 for each group, were enrolled. One patient in the OFD group discontinued early, and analysis was performed on 18 patients of the REG group (12 females and 6 males; mean age, 55.9 ± 9.2) and on 17 patients of the OFD group (10 females and 7 males; mean age, 58.3 ± 11.6). Demographic and clinical data of patients are reported in Table 1, and no significant differences were found between the groups. Uneventful wound healing occurred in all the operated sites. In the sites treated with regenerative procedure, no membrane exposure occurred, and all membranes were removed at 5-6 weeks after surgery.

### 3.1. CEMP-1 Levels

**Figure 4:**
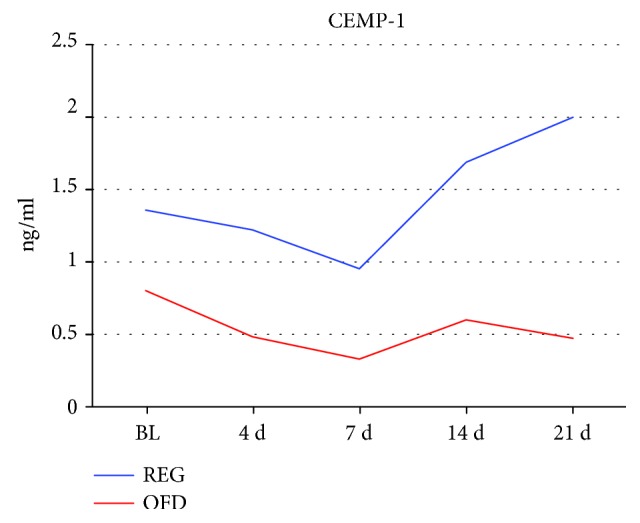
Levels of CEMP-1 in gingival crevicular fluid (at baseline, 0) and periodontal wound fluid at 4, 7, 14, and 21 days (d), postop in sites treated with regenerative approach (REG) and open flap debridement (OFD).

Levels of CEMP-1 in gingival crevicular fluid at baseline and periodontal wound fluid at 4 days and 1, 2, and 3 weeks postop in the sites treated with regenerative approach and open flap debridement are reported in Figure 4.

At baseline, CEMP-1 showed significantly different levels between the REG and OFD groups (OFD mean 0.80, REG mean 1.39; *t*-test, *p* = 0.035); variability was also different between the two groups (OFD variance 0.46, REG variance 0.78).

Due to these differences at baseline, direct comparisons of CEMP-1 at later time points would have been inappropriate, to make up for this bias a *t*-test was performed on the difference from baseline CEMP-1 values between the groups at day 21st. This test was also significant (*p* = 0.031), with the REG group recording a 0.64 ng/ml rise in CEMP-1, and the OFD a 0.32 ng/ml fall between baseline and day 21st. Finally, to determine whether the change from baseline within each group was significant, pairwise *t*-tests were performed on the REG and OFD groups between baseline and day 21st individually. These tests indicated that the change in the REG group, even though larger (+0.64 ng/ml) was not statistically significant (*p* = 0.12), while the smaller change in the OFD group (-0.32 ng/ml) was barely significant (*p* = 0.049), this result underscores the difference in variance between the 2 groups.

**Figure 5:**
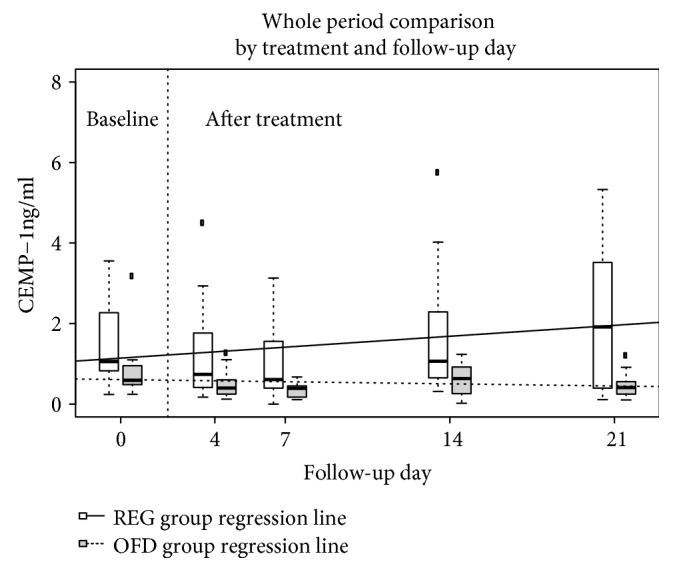
Plot representing distribution of CEMP-1 in ng/ml by treatment and week (baseline is day 0, while days 4 to 21 are after treatment) using box plots. The REG group is white, OFD is grey, the thick black line is the median, the box contains the 1st to the 3rd quartile, and the whiskers are the lowest and highest values within 1.5 times the interquartile range from the box, other values are represented as outliers. The regression lines from the linear mixed model are also plotted (REG, line; OFD, dashed line).

Figure 5 represents the data by timepoint (baseline and 21 days after intervention) and by the treatment group using boxplots. On inspection, strong differences in CEMP-1 levels emerged, both in value and in variability. The REG group CEMP-1 average and median values are higher than the OFD group ones at all time points, but also noticeably more dispersed as shown in the descriptive and preliminary analysis above. The model confirmed what can be seen in the data from inspection i.e., (1) that there was a significant average difference in CEMP-1 values between the REG and OFD groups at baseline (*p* = 0.041), the CEMP-1 modeled average in the OFD group was lower by 0.45 ng/ml, (2) there was a significant trend in CEMP-1 over time and this trend was different among the 2 groups: the REG group showed a statistically significant rising CEMP-1 trend (0.18 ng/ml a week *p* = 0.012), while the OFD had a trend that was significantly lower (-0.22 ng/ml a week compared to the REG group trend *p* = 0.023), the OFD group lost on average 0.05 ng/ml a week.

### 3.2. Analysis of Correlation

A significant correlation (Spearman correlation) was found in the REG sites among

- CEMP-1 levels at 3 weeks and probing pocket depth reduction ((*p* = 0.041, *r* = 0.486), (*p* = 0.801 in the OFD sites))
- differential CEMP-1 levels between 3 w and baseline and probing pocket depth reduction ((*p* = 0.041, *r* = 0.485), (*p* = 0.128 in the OFD sites))
- differential CEMP-1 levels between 3 w and baseline and probing pocket depth baseline ((*p* = 0.027, *r* = 0.519), (*p* = 0.102 in the OFD sites))

## 4. Discussion

During the early (inflammatory) phase of healing that occurs after periodontal surgery, the levels of inflammatory markers peak, thus negatively affecting periodontal as well as cementum neoformation and shifting healing toward a more reparative than regenerative process [8, 15]. In periodontal regenerative surgery, accuracies are put in place to achieve regeneration of periodontium (alveolar bone and cementum with inserted perpendicularly oriented periodontal ligament fibers) and the trend of regenerative markers may be affected accordingly. In the present study, we evaluated if the levels of CEMP-1 in periodontal wound fluid change during initial healing after surgical procedure designed to regenerate periodontium. From data, it resulted a different trend of PWF CEMP-1 amount after periodontal regenerative surgery compared to periodontal surgery alone. In the REG sites, after initial decrease, the protein level increased significantly, while in the OFD sites remained at levels significantly lower than baseline. The decreased amount of this protein observed in all the treated sites after surgery may have resulted from the activation of inflammatory cells, in the initial healing phase [8]. Initial defect degranulation and subsequent removal of cells that synthesize CEMP-1 may also have delayed the production of proregenerative proteins as well as CEMP-1. In the OFD sites, the sustention of low levels of the analyzed protein may indicate the ongoing periodontal tissue repair with limited formation of new cementum/mineralized tissue. After OFD, the recruitment and activation of cells may mainly be devoted to epithelium and connective tissue healing as supported by increased amounts of fibroblast growth factor-2 and transforming growth factor-*β* [1]. On the contrary, in the REG sites, the regenerative procedure seems to invert this tendency increasing the CEMP-1 secretion by cementoblasts; the mineralization activity seems to resume after the initial flexion, thus suggesting the beginning of the cementum regeneration with insertion of periodontal ligament fibers. These data on different amounts of protein for tissue neoformation confirm those of previous study. A different trend of local levels of matrix metalloproteinase-1 and bone morphogenetic protein-7 during early wound healing was found between periodontal surgery associated with regenerative procedure and periodontal surgery alone. Furthermore, this data was found to be related with the clinical outcome of periodontal regenerative surgery, while sites that underwent open flap debridement showed no such association, thus suggesting that the connective tissue neoformation and remodelling was ongoing at the site treated with regenerative therapy [5].

CEMP-1 has been analyzed in the present study as a tissue-specific protein for cementum. This protein is expressed by cementoblastoma-derived cells and periodontal ligament cells and is localized throughout the cementum [6]. CEMP-1 seems to play a role in the early phases of mineralization by promoting the formation of apatite crystals [6]. Within the periodontal ligament, this protein stimulates the recruitment, proliferation, and maturation of mesenchymal stem cells promoting their mineralization activity [16]. Data on class II furcation defects of monkeys treated with Matrigel® matrix alone or associated with human transforming growth factor-*β*3 demonstrated that this growth factor induces cementogenesis by upregulation of CEMP-1 and that CEMP-1 is an indicator of cementogenesis [17].

The correlation analysis has shown that the increase of CEMP-1 from baseline in the REG sites is moderately but significantly related to PD reduction at 6 months. It can be speculated that the short-term clinical outcome of periodontal regenerative therapy may be predicted by early molecular analysis of wound fluid. The sites with intrabony defects treated with the regenerative approach showed significantly higher levels of CEMP-1 than OFD sites both at baseline and at all postop appointments, and the probing pocket depth at baseline was related to the increase of CEMP-1 from baseline to 3 weeks. Periodontal defects with a vertical bone component have higher physiological remodeling activity and innate regenerative potential than those with suprabony component, and this may induce higher basal levels of CEMP-1. For ethical reason, it was not possible to apply the same surgical protocol to infrabony and suprabony defects. The two interventions selected for this trial are different in their nature, surgical protocol, and expected periodontal healing events (regeneration vs. repair). In the sites where the primary goal of intervention was tissue regeneration, it may be expected that the blood clot stabilized under the barrier membrane is progressively replaced by a new bone, periodontal ligament, and cementum, as demonstrated histologically in humans [18]. Otherwise, in the sites treated with OFD where the primary goal was the pocket reduction without tissue regeneration, no relevant cementogenesis or osteogenesis is expected, and human histological studies reported the formation of long junctional epithelium with parallel-oriented collagen fibers [19, 20]. On the base of these different healing profiles, the postop higher levels of CEMP-1 found in the REG sites than those in the OFD sites may reflect the stronger cementogenesis and tissue mineralization that occur in infrabony defects after regenerative procedure.

Ethical limits of harvesting periodontal tissue after regeneration in patients make the research of alternative noninvasive methods to assess biological events that occur during healing necessary. In clinical practice, evaluation of cementogenesis by analysis of wound fluid may be useful to predict the success of periodontal regeneration as well as to support the development of further regenerative approaches. To our knowledge, the present study is the first to define the possible use of CEMP-1 as an early predictor of clinical outcome and as a marker for cementogenesis, by confirming its presence in gingival crevicular fluid/periodontal wound fluid of patients and finding increased levels of this protein mostly in the sites where regeneration of periodontal complex was expected. Suprabony defects were chosen for comparison since their different healing models supposedly induce a limited CEMP-1 production thus highlighting the role of this protein during healing. As the last point, it is necessary to identify the reference level over which periodontal regenerative therapy can be defined as successful. This reference level may be hardly deductible, due to the high variability in CEMP-1 levels found especially in the REG group. This data that may reflect the interindividual variability in regenerative potential that normally exists between patients, as well as the intraoperator variability for surgical performances. For these reasons, further studies with increased number of samples and histological confirmation in humans are needed.

The application of this protein as an agent promoting cementum regeneration would also be interesting. The effects of bioresorbable scaffold loaded with rhCEMP-1 on the attachment, proliferation, and osteogenic and cementogenic differentiations of human periodontal ligament cells have been investigated histologically in a critical size defect on rodent, and the potential of generating cementum-like tissue in vitro and in vivo has been demonstrated [21].

## 5. Conclusions

Data from this study supports that, after surgery, during the initial inflammatory phase of periodontal healing, CEMP-1 levels decrease regardless of the surgical protocol applied. The surgical procedures used to regenerate periodontal tissue are able to reverse this trend and to induce significant increase of CEMP-1 in periodontal wound fluid after the first week postop.

Limited to gingival crevicular fluid/periodontal wound fluid, CEMP-1 could be a marker for possible ongoing cementogenesis during the first 21 days of healing. It would be interesting to design further clinical and histological studies evaluating levels of this protein in tissue and in crevicular fluid of healthy sites as well as after different nonsurgical/surgical procedures with the purpose to define a reference value that may discriminate between healing patterns.

## Figures and Tables

**Table 1:** Data on study population.

Parameter	Mean ± sd in the REG group	Mean ± sd in the OFD group	*P* value between the groups
Age (years)	55.9 ± 9.2	58.3 ± 11.6	ns

Full mouth plaque score (%)	5.7 ± 2.8	6.5 ± 2.9	ns

Full mouth bleeding score (%)	3.9 ± 1.8	4.6 ± 2.9	ns

Probing pocket depth at experimental sites (mm)	8 ± 1.8 (BL)	5.3 ± 0.6 (BL)	ns
	4.1 ± 1 (6 m)	2.9 ± 0.7 (6 m)	

Clinical attachment level at experimental sites (mm)	9.7 ± 2.9 (BL)	6.2 ± 1.5 (BL)	ns
	5.4 ± 2.1 (6 m)	4.3 ± 1.1 (6 m)	

Experimental sites that bleed on probing at baseline (%)	61.1% (BL)	44.4% (BL)	ns
	5.5% (6 m)	0 (6 m)	

REG: sites that underwent to regenerative therapy; OFD: sites treated with open flap debridement; NS: not statistically significant; BL: baseline; 6 m: 6 months.

## Data Availability

The molecular and clinical data used to support the findings of this study are included within the article.
